# Eco-friendly method for the analysis of the soluble fraction of parabens leached from sludge of a hospital wastewater treatment plant—proof of concept

**DOI:** 10.1007/s00216-026-06762-1

**Published:** 2026-10-08

**Authors:** S. Bedoya Patiño, J. P. Arrubla Vélez, J. M. Grisales Bedoya, M. Becerra-Herrera, P. Richter, E. Carasek, J. J. Ríos Acevedo, M. Rosero-Moreano

**Affiliations:** 1 Facultad de Ciencias Básicas y Tecnologías, Universidad del Quindío, 630004 Armenia, Colombia; 2 Grupo de Investigación en Cromatografía y Técnicas Afines GICTA, Departamento de Química, Facultad de Ciencias Exactas y Naturales, Universidad de Caldas, Calle 65 No 26-10, 170004 Manizales, Colombia; 3 Facultad de Tecnología, Universidad Tecnológica de Pereira, 660003 Pereira, Colombia; 4 Departamento de Química, Facultad de Ciencias Experimentales, Universidad de Huelva, Huelva, Spain; 5 Department of Inorganic and Analytical Chemistry, Faculty of Chemical and Pharmaceutical Sciences, University of Chile, P.O. Box 233, 9170124 Santiago, Chile; 6 Departamento de Química, Universidad Federal de Santa Catarina, Florianópolis, 88000-000 Brasil; 7 Facultad de Ciencias Básicas, , Universidad Santiago de Cali, Calle 5 No 62-00, 76036 Cali, Colombia

**Keywords:** Parabens, Sludge, Bioavailable fraction, SPME, RDSE, GC-MS, On-fiber derivatization

## Abstract

Developing eco-friendly analytical methods is crucial for monitoring emerging pollutants in complex matrices. This study presents a methodology based on green chemistry for determining the bioavailable fraction of methylparaben (MeP), ethylparaben (EtP), propylparaben (PrP), and butylparaben (BuP) in hospital wastewater treatment plant sludge. The approach combines in situ leaching with rotating-disk sorptive extraction (RDSE) in reverse mode, followed by direct-immersion solid-phase microextraction (DI-SPME) and headspace (HS-SPME) with on-fiber derivatization. GC-MS analysis identified the DVB/CAR/PDMS fiber as the most effective due to its high analyte affinity. Derivatization was successfully performed at room temperature in 10 min, with mass spectral match factors above 85%. The active analytical workflow (SPME extraction, on-fiber silylation, and GC-MS analysis) is completed in under 60 min per sample, enabling high-throughput screening once the parallel overnight leaching is completed. The method shows high sensitivity (LODs: 2.1–5.2 ng/g), good reproducibility (RSD < 9%), and accuracy (recoveries: 80–96% at 800 ng/g, *n* = 3). The four parabens were quantified in real sludge samples; MeP, EtP, and BuP were detected below their limits of quantification (< LOQ), while PrP was quantified at 20.0 ± 2.4 ng/g. Significant matrix effects (50% signal reduction) necessitated a standard additions protocol. The method’s greenness, practicality, and innovation were validated using AGREEprep (score 0.54), BAGI (score 75.0), and VIGI (score 75) metrics, confirming its high sustainability, routine feasibility, and innovative character for complex environmental sample preparation.

**Graphical Abstract:**

**Figure:**
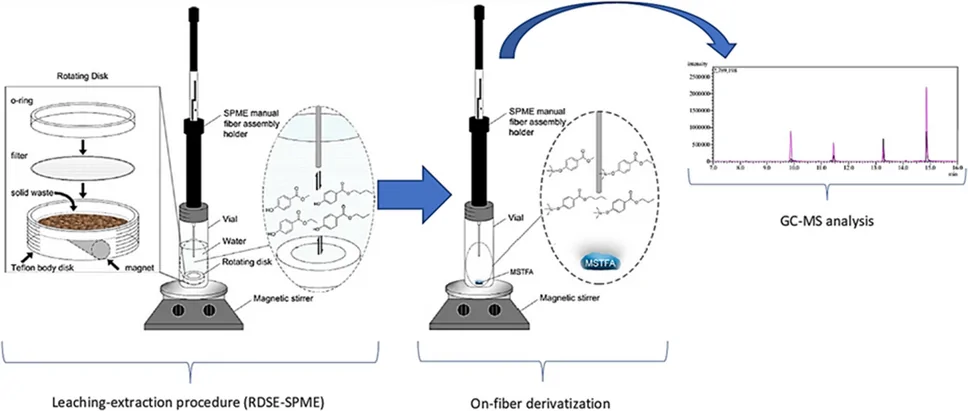

**Supplementary Information:**

The online version contains supplementary material available at https://doi.org/10.1007/s00216-026-06762-1.

## Introduction

Parabens are made up of ester groups derived from p-hydroxybenzoic acid, and they differ in the alkyl group chain length. Parabens are widely used as preservatives in food, personal care products, and pharmaceutical preparations [1–3]. They are classified as emerging pollutants with endocrine-disrupting potential, and they result in adverse effects on both the environment and human health [1, 4]. It is speculated that parabens may influence the growth and development of malignant tumors or melanomas [5–7]. Parabens are present in approximately 80% of personal care products [4]. A study evaluating the flow of paraben-containing substances in Denmark reported that 36% of the 751 products were considered to contain parabens [8]. A Norwegian study revealed that parabens were present in 32% of 117 baby care products. Another estimate shows that butylparaben is present in 13% of cosmetics and personal care products, while propylparaben and/or methylparaben is present in 48% of these products [4]. In the USA, the paraben content in daily cosmetic products was estimated to be 17.76 g for adults and 378 mg for infants [9]. One study estimated the concentration of parabens in human urine samples from various Asian countries, Greece, and the USA, confirming that methylparaben and ethylparaben are the parabens with the highest concentrations (mean, µg/kg body weight/day): China (3.67), India (1.36), Japan (1.89), Korea (34.2), Kuwait (1.21), Saudi Arabia (1.60), Vietnam (0.62), Greece (1.02), and the USA (0.88) [10].

The longer the chain of the ester group is, the greater the antimicrobial activity; however, the solubility in water decreases [11]. The chemical structure of parabens, featuring an ester group and a phenolic hydroxyl group, promotes both hydrogen-bond donor and acceptor properties. Their moderate water solubility and affinity for organic phases are well characterized by their octanol-water partition coefficients (*log K_ow* ranging from 1.96 to 3.57, Table 1).

**Table 1:** Chemical characteristics of the target parabens [4, 11]

Analyte	Chemical formula	Molecular weight (g/mol)	Water solubility (25 °C, g/100 mL)	pKa	CAS number	Log P
MeP	C_8_H_8_O_3_	152.16	2.00	8.17	99-76-3	1.96
EtP	C_9_H_10_O_3_	166.18	0.86	8.22	120-47-8	2.47
PrP	C_10_H_12_O_3_	180.21	0.30	8.35	94-13-3	3.04
BuP	C_11_H_14_O_3_	194.23	0.15	8.37	94-26-8	3.57

Sewage sludge from wastewater treatment plants (WWTPs) is a hazardous waste due to its high content of emerging pollutants, including parabens. Therefore, it must be subjected to stabilization processes to reduce its pollutant load and ultimately obtain a product called biosolids. Biosolids can be used as organic fertilizer, a substrate for regenerating contaminated soils, or artificial soil in construction, making them economically beneficial [12]. It is crucial to determine the bioavailable fraction of these pollutants in sludge, as this represents the portion that can be effectively leached and released into the environment upon land application.

In 2009, Richter P. et al. developed a Teflon rotary disk for extraction as an alternative to stir bar sorptive extraction (SBSE) to enable higher stirring speeds; this technique is called rotating-disk sorptive extraction (RDSE) [13]. The disk has a cavity to contain an adsorbing solid phase; this cavity is covered with a fiberglass filter and sealed with a Teflon ring [14–16]. Our research group has extensive experience with the analysis of parabens from wastewater using RDSE and GC-MS [17]. In this context, the proposed method differs fundamentally from our previous work in water analysis (e.g., Becerra-Herrera et al. [17]). In the current study, the RDSE device is operated in a “reverse mode” to function as an in situ solid-liquid leaching device. The solid sludge sample is placed inside the disk’s cavity, and water is allowed to permeate it, simulating the release of the soluble, bioavailable fraction of contaminants. This represents a novel conceptual mechanism of leaching and release simulation rather than conventional sample extraction and clean-up.

Commonly, determining paraben concentration in sludge involves liquid-liquid extraction followed by solid-phase extraction (SPE) or matrix solid-phase dispersion (MSPD), requiring considerable time, toxic organic solvents, and specialized reagents. A more eco-efficient technique like SBSE with in situ derivatization has been used for soils but requires additional wash steps prior to thermal desorption [18]. The combination of RDSE in reverse mode and solid-phase microextraction (SPME) reduces procedural errors and solvent use, making it an innovative approach for the analysis of the bioavailable fraction of parabens in solid samples [19].

Since environmental monitoring has mainly focused on dissolved parabens in water, only limited studies address parabens in residual sludge [20, 21]. This is due to matrix complexity, requiring selective techniques like LC-MS/MS or GC-MS/MS [22]. Despite its benefits, GC has been used sparingly for parabens due to the need for derivatization [17–19, 23]. On-fiber derivatization is a practical, green alternative where the fiber is impregnated with the derivatizing agent or exposed to its vapors in a closed vial [24, 25]. While liquid chromatography-tandem mass spectrometry (LC-MS/MS) is frequently preferred in recent literature for paraben analysis in sludge to avoid derivatization, GC-MS remains a highly attractive alternative. GC-MS is significantly more cost-effective, widely available in routine laboratories, and provides electron ionization (EI) mass spectra with highly specific fragmentation patterns. When coupled with on-fiber derivatization, GC-MS overcomes its main limitation—analyte polarity—without requiring large solvent volumes, offering a practical and accessible tool for complex matrices.

The aim of this work is to develop an alternative analytical methodology based on green chemistry to evaluate the bioavailable fraction of parabens in real samples of a hospital WWTP sludge. This leverages our originally developed RDSE technique in reverse mode in conjunction with SPME to provide a simple, green, and efficient analysis using GC-MS.

## Experimental

### Materials and reagents

Water (18.2 MΩ cm) was purified using a Millipore Milli-Q system (Bedford, MA, USA). methylparaben (MeP), ethylparaben (EtP), propylparaben (PrP), and butylparaben (BuP) were purchased from Sigma Aldrich. 2,2,2-Trifluoro-N-methyl-N-(trimethylsilyl)acetamide (MSTFA) was supplied by Merck. Analytical-grade ethyl acetate was used for standard solutions, and nitrogen gas (N_2_) grade 5.0 was applied for concentration.

SPME tests were performed using a commercial manual holder, a 50/30 µm divinylbenzene/carboxen/polydimethylsiloxane (DVB/CAR/PDMS) fiber, and an 85 µm polyacrylate (PA) fiber from Supelco (Bellefonte, PA, USA). Fibers were conditioned prior to use.

### Sludge sampling, conditioning, matrix clean-up, and spiking procedure

Sludge samples were collected from a wastewater treatment plant (WWTP) at the Santa Sofia Hospital (Manizales, Colombia), which operates with an activated sludge system and a bacterial consortium (Septitrim®). For sample conditioning, the collected sludge was dried at room temperature for 7 days, macerated, sieved (< 1 mm and 50 µm), and stored in amber vials at 4 °C. Total organic carbon (TOC) was measured using a TOC Teledyne Tekmar Combustion Analyzer. To obtain a clean, interference-free sludge matrix for subsequent recovery and validation studies, an exhaustive extraction protocol was performed: the dried sludge was extracted with 10 mL of dichloromethane (four times) and 5 mL of methanol (four times) in an ultrasonic bath (30 min each). The cleaned sludge was then placed in a desiccator for 48 h to fully evaporate solvent residues, and any remaining agglomerations were carefully eliminated [16]. The matrix parameters were determined to be pH 7.2 in aqueous suspension, a residual moisture content of 1.8 ± 0.2%, and a sieved particle size fraction of < 50 µm for the cleaned matrix. It is important to emphasize that this aggressive cleaning protocol was performed solely to prepare a blank matrix for recovery and calibration validation due to the lack of certified reference materials (CRMs) for parabens in sewage sludge. This cleaning procedure is not part of the routine analytical workflow. We acknowledge that re-spiking cleaned sludge does not fully replicate the long-term chemical aging and sequestration of native parabens in real-world sludge (aging effect). For the preparation of spiked sludge samples, a 125 µg/g standard solution of parabens in ethyl acetate was prepared; then, 0.5849 g of the clean, dry sludge was added to an aliquot of this solution and stirred for 1 h. The organic solvent was evaporated under a gentle nitrogen stream to obtain spiked dry-weight concentrations, and all recovery and validation analyses were performed in triplicate (*n* = 3) (Fig. S1, Electronic Supplementary Material [ESM)].

### Hyphenated RDSE-SPME leaching, extraction, and on-fiber derivatization procedure

**Fig. 1:**
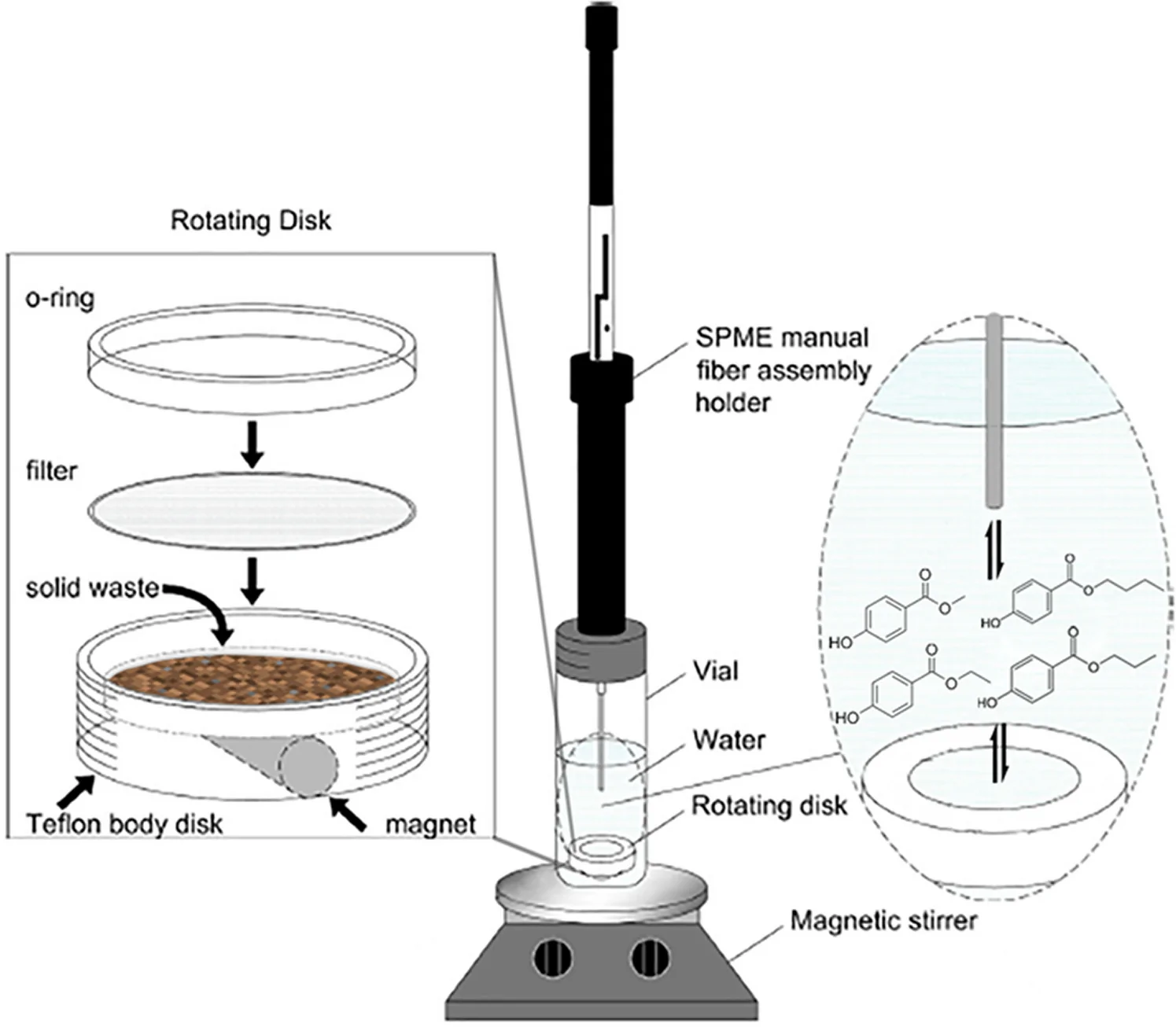
Schematic diagram of simultaneous SPME and rotating-disk desorption Adapted from Mendes et al. [16]

The integrated RDSE-SPME extraction system comprised two main devices. For the rotating-disk sorptive extraction (RDSE) in reverse mode, rotating Teflon disks (1.5 cm diameter) containing an internal magnetic stirring bar were used. These disks feature a 0.44 cm^3^ cavity where 40 mg of sieved sludge sample was deposited, which was subsequently covered with a fiberglass filter paper (0.2 µm pore size) and sealed with a Teflon ring [16]. For the solid-phase microextraction (SPME) step, a commercial manual holder was used to support the fibers. To compare extraction efficiencies during method development (Fig. 1), a 50/30 µm divinylbenzene/carboxen/polydimethylsiloxane (DVB/CAR/PDMS) fiber and an 85 µm polyacrylate (PA) fiber from Supelco (Bellefonte, PA, USA) were evaluated. The selection of these two fibers was based on their polarities and suitability for direct immersion. The DVB/CAR/PDMS coating is a bipolar sorbent that combines absorption and adsorption mechanisms, making it highly efficient for polar analytes and their silylated derivatives. Other coatings, such as pure PDMS, were excluded due to their low affinity for polar parabens. This evaluation was conducted in a 5 mL vial containing 4 mL of Milli-Q water spiked with 100 µL of a 1000 µg/g analyte solution, where the fibers were exposed via direct immersion for 30 min at 1300 rpm, followed by on-fiber derivatization and GC-MS analysis.

**Fig. 2:**
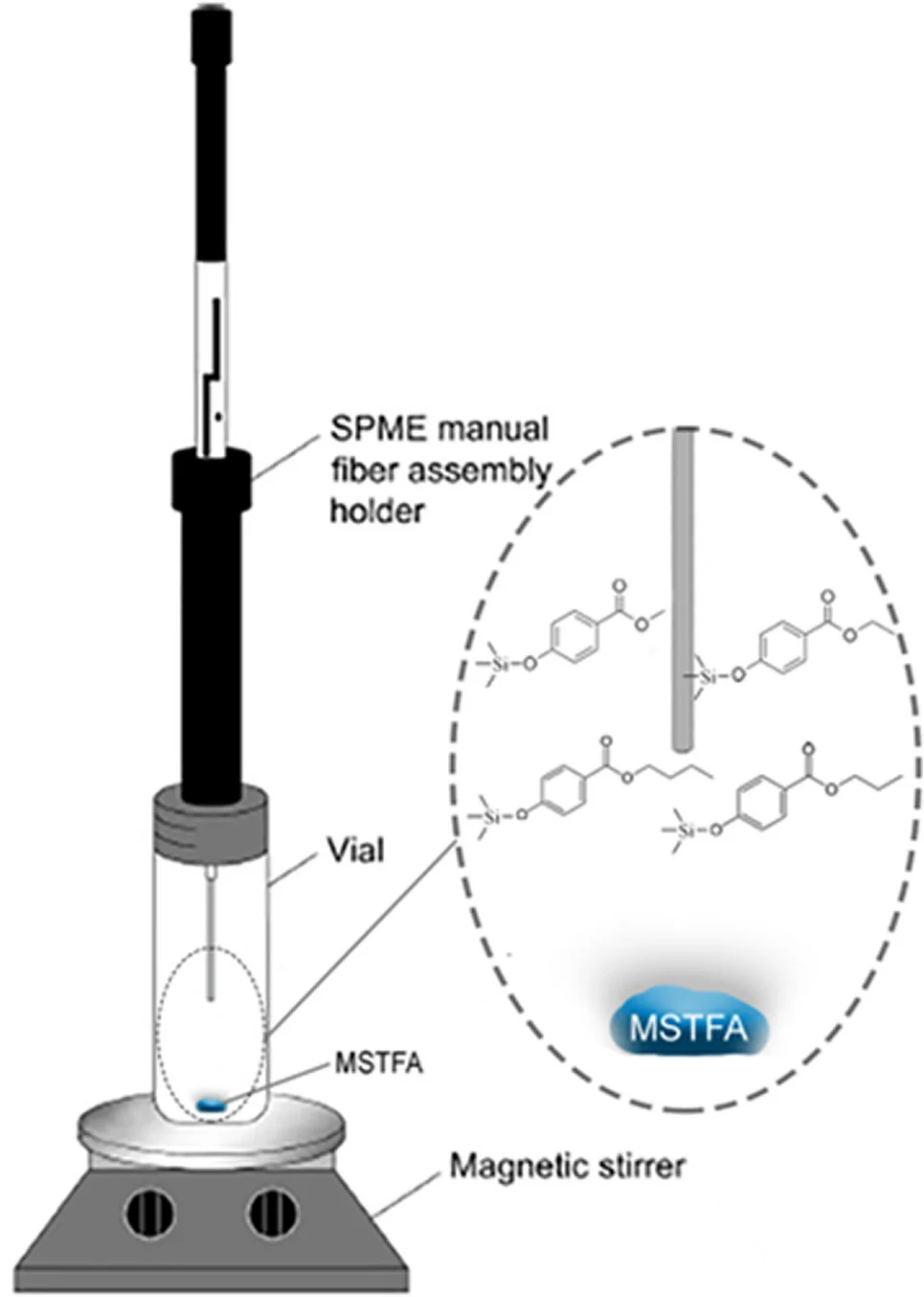
Schematic diagram of on-fiber derivatization with MSTFA Adapted from Mendes et al. [16]

For the routine leaching-extraction workflow, the rotating disk was pre-cleaned by sonication in acetone and dried. Once the 40 mg sludge sample was loaded and the disk sealed, it was placed in a vial containing 25 mL of Milli-Q water and stirred at 1500 rpm overnight (approximately 12 h) to simulate the natural release of the highly mobile water-soluble fraction under aggressive leaching conditions, which represents the most readily bioavailable fraction upon rainfall or agricultural land application. Using Milli-Q water rather than aggressive regulatory fluids represents a more realistic simulation of natural agricultural runoff. While Milli-Q water serves as a standardized leaching phase, we note that in real-world scenarios, leaching fluids would contain dissolved organic matter (DOM) and inorganic ions, which could influence leaching kinetics and equilibrium. The pH of the aqueous phase was monitored before and after the overnight leaching, remaining stable between 6.8 and 7.1. Since the pKa of all four parabens is above 8.1 (Table 1), they remain entirely in their neutral, non-ionized forms within this pH range, ensuring stable extraction efficiency. Following this leaching stage, the DVB/CAR/PDMS SPME fiber was exposed directly to the aqueous phase via direct immersion for 30 min at 1500 rpm to extract the leached parabens. Immediately after the extraction, the fiber was retracted, gently dried with a lint-free tissue for 5 s to remove adhering water droplets, and then transferred to the headspace of a 1.5 mL vial containing 20 µL of MSTFA, where on-fiber silylation was carried out for 10 min at room temperature before thermal desorption at 250 °C in the GC-MS injection port (Fig. 2).

### Instrumentation and GC-MS analysis

A GCMS-QP-2020 Shimadzu equipped with a 30 m × 0.25 mm × 0.25 µm SH-Rxi-5Sil MS column was used. Conditions: splitless injection, initial oven temperature of 75 °C (hold 1 min), rate of 20 °C/min to 150 °C (hold 5 min), and 10 °C/min to 300 °C (hold 5 min). Helium (grade 5.0) flow was 1.0 mL/min. Injection port and interface temperatures were 250 °C; ion source was 220 °C. The SPME fibers were desorbed at 250 °C for 10 min. MS operated in EI mode (70 eV) using full scan (50–510 m/z) and Selected Ion Monitoring (SIM) mode (193, 195, 209, 210, 223, and 224 m/z). Identification was confirmed using the NIST 14 library.

### Method validation and greenness, practicality, and innovation evaluation

Due to the lack of a commercially available certified reference material (CRM) for parabens in hospital WWTP sludge, the accuracy of the method was evaluated using a surrogate matrix. This consisted of clean sludge, completely free of parabens, prepared through exhaustive solvent extraction (as described in “Sludge sampling, conditioning, matrix clean-up, and spiking procedure”), which was subsequently spiked with known concentrations of the target analytes. The RDSE-SPME-GC-MS method validation was rigorously performed using sewage sludge as a matrix. To ensure robustness, repeatability, and linearity, and to compensate for the significant matrix effects, a standard additions calibration protocol spanning from 10 to 1000 ng/g (dry weight) was prepared daily and analyzed in duplicate over 5 consecutive days. Due to the absence of suitable and affordable isotope-labeled internal standards, standard additions calibration was performed on a sample-by-sample basis to mathematically correct for matrix effects. Analytical sensitivity was established using the IUPAC criteria, yielding limits of detection (LOD) at a signal-to-noise ratio (S/N) of 3 (ranging from 2.1 to 5.2 ng/g) and limits of quantification (LOQ) at S/N = 10 (~ 10 to 20 ng/g). The LOD and LOQ were calculated from the signal-to-noise ratio of 3 and 10, respectively, using 10 blank matrix extracts (*n* = 10) and measuring the peak-to-peak noise in a 1-min window adjacent to the analyte retention time. Method accuracy was successfully validated using raw, uncleaned WWTP sludge by subtracting the mean endogenous background concentration determined from unspiked control replicates (*n* = 3) from the total concentration measured in spiked raw sludge replicates (*n* = 2, at 800 ng/g). To provide a comprehensive evaluation of the developed methodology, its environmental sustainability, routine practicality, and innovative character were quantitatively assessed using three complementary metric tools: the Analytical Greenness Metric for Sample Preparation (AGREEprep) [26], the Blue Applicability Grade Index (BAGI) [27], and the recently introduced Violet Innovation Grade Index (VIGI) [28]. Details on the scoring parameters for all metrics are provided in Tables S1–S3 of the Electronic Supplementary Material (ESM). AGREEprep focuses on the environmental impact of sample preparation (scale 0–1). BAGI evaluates the operational practicality and routine applicability of the method based on 10 attributes (scale 25–100, where a score ≥ 60 indicates a practical method). VIGI evaluates the technological and conceptual innovation of the method based on 10 specific parameters (scale 0–100, where a score ≥ 50 indicates an innovative method), generating a visual star-shaped decagon pictogram.

## Results and discussion

### Optimization of the extraction and derivatization parameters

To ensure the reliable determination of the bioavailable fraction of parabens, the conditions related to the rotating disk extraction and derivatization were carefully established, building upon the principles of our previously developed RDSE methodologies [13, 15, 17]. The mass of the sludge sample placed in the disk was fixed at 40 mg to fit optimally within the 0.44 cm^3^ cavity of the disk without causing saturation or restricting fluid flow through the filter. The stirring speed was set to 1500 rpm to maximize mass transfer of the analytes from the solid matrix into the aqueous medium without compromising the physical integrity of the disk. An overnight leaching period was employed to guarantee the exhaustive release of the bioavailable fraction of parabens from the highly complex sludge matrix. The physical integrity of the 0.2 µm fiberglass filter was verified after the overnight agitation, showing no mechanical breakdown or particulate leakage. The turbidity of the aqueous phase was measured (using a portable turbidimeter) and found to be negligible, ensuring that only the truly dissolved, bioavailable fraction was subjected to SPME extraction. On-fiber derivatization using MSTFA was performed using 20 µL of MSTFA in a sealed 1.5 mL vial. A 10-min exposure at room temperature was adequate to derivatize the extracted parabens on the SPME fiber efficiently, achieving high silylation yields with mass spectral match factors above 85% against the NIST 14 database, without the need for external heating.

### Fiber evaluation

**Fig. 3:**
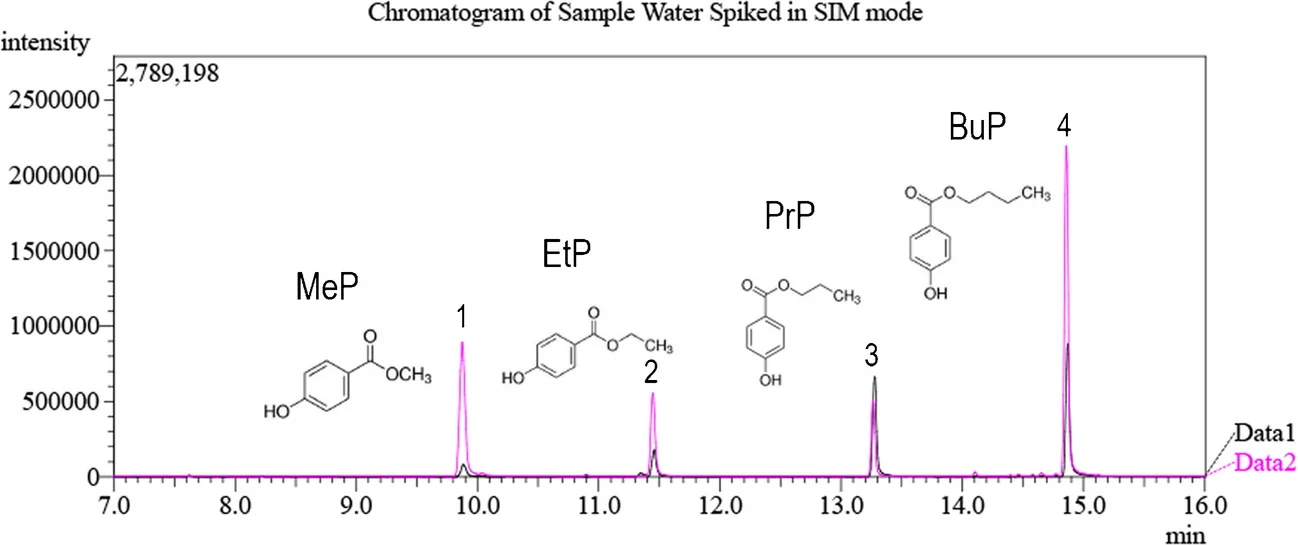
SIM mode chromatogram of a water sample spiked with parabens extracted from different fibers: (Data 1) PA; (Data 2) DVB/CAR/PDMS. Extraction conditions: direct immersion for 30 min at 1300 rpm

The DVB/CAR/PDMS fiber generated signals with significantly greater intensity than the PA fiber, indicating a superior extraction efficiency for all four parabens under the proposed conditions. The mixed stationary phase provides an excellent affinity for compounds of varying polarities, making it the most suitable choice for subsequent analyses (Fig. 3).

### Extraction kinetics and thermodynamic principles of SPME

**Fig. 4:**
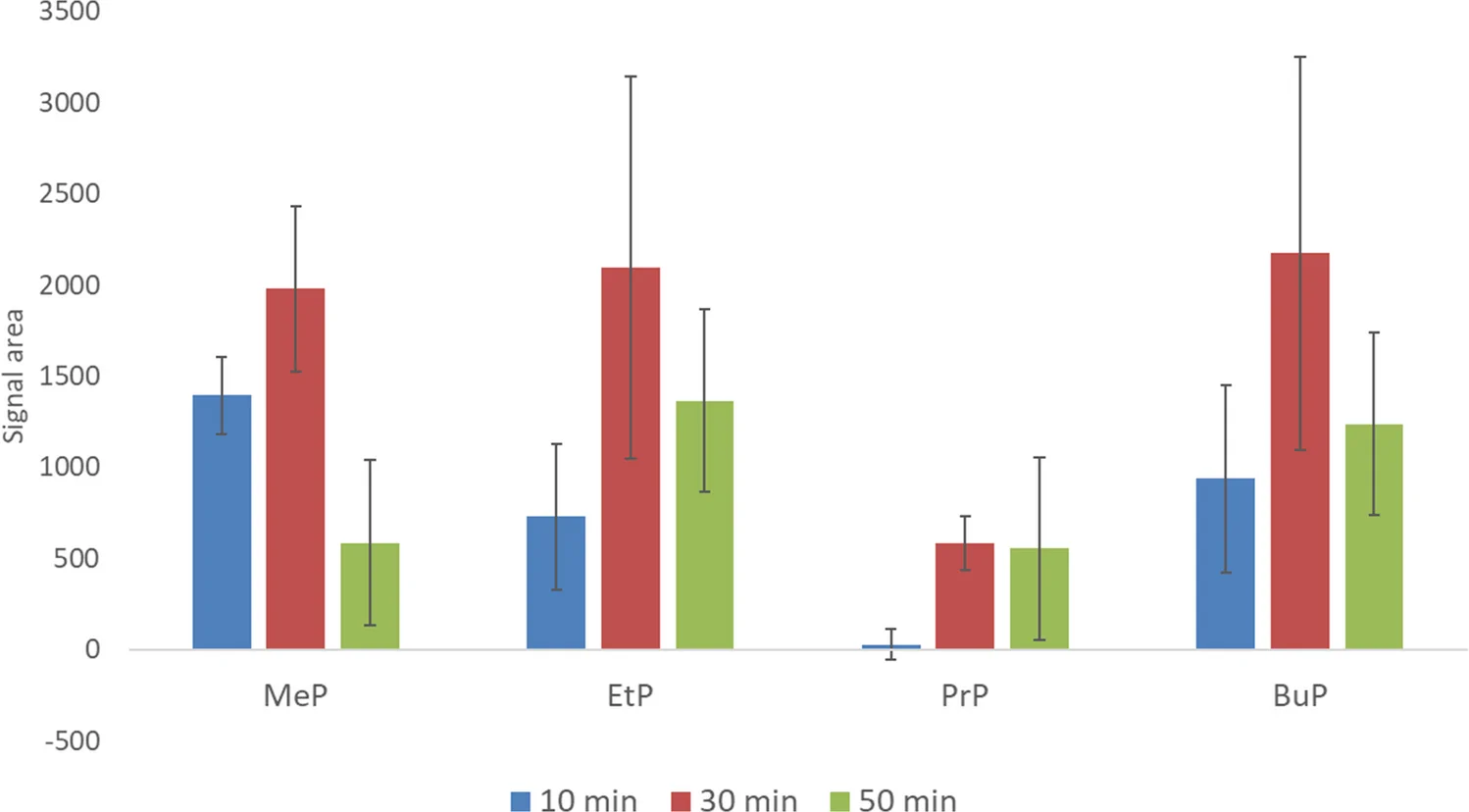
Optimization of SPME extraction time (10, 30, and 50 min) using DVB/CAR/PDMS fiber. Agitation speed: 1500 rpm. Error bars represent standard deviations (*n* = 3)

The extraction kinetics and thermodynamic behavior of the RDSE-SPME process were evaluated to understand the partitioning of the parabens onto the DVB/CAR/PDMS fiber coating, as illustrated in the extraction profiles shown in Fig. 4. Solid-phase microextraction is a thermodynamics-driven process. Under our experimental conditions, where the sample volume (*V𝓈* = *25* mL) is significantly larger than the volume of the thin polymeric coating (*V𝒻 ≈ 0.5* µL), the term *V𝓈 ≫ K𝒻s V𝒻* holds true, and the equilibrium equation simplifies to: *n* = *K𝒻s V𝒻 C₀*, where *n* is the amount of analyte extracted, *K𝒻s* is the partition coefficient, and *C₀* is the initial concentration. This relationship highlights that the amount of analyte extracted is directly proportional to its concentration, independent of the sample volume.

The optimization profiles (Fig. 4) demonstrate that the extracted amounts increase during the first 30 min, representing the kinetic phase, and approach a practical equilibrium. A slight decrease in peak areas was observed at 50 min for the lighter parabens (MeP and EtP), which is attributed to competitive displacement on the adsorption sites of the DVB/CAR/PDMS fiber by the more hydrophobic, heavier parabens (PrP and BuP). The thermodynamic affinity follows the *log K_ow* values (MeP = 1.96, EtP = 2.47, PrP = 3.04, BuP = 3.57), although the absolute peak areas are also influenced by MS sensitivity for the selected quantifier ions. The standard deviations are associated with the manual handling of the fiber. Furthermore, the energetic agitation of 1500 rpm provided by the RDSE device accelerates the extraction kinetics, reducing the boundary layer thickness at the fiber interface and facilitating rapid extraction within a practical 30-min window.

### Evaluation of the RDSE-SPME-GC-MS method

The analytical features of the proposed methodology were evaluated under optimized conditions. Due to the significant matrix effects observed in the sewage sludge matrix (resulting in approximately 50% signal suppression (Table 2)), a standard additions calibration protocol was utilized to establish linearity.

**Table 2:** Ratio of selected ion monitoring (SIM) signals in cleaned vs. raw hospital WWTP sludge

Compound	Signal ratio (m/z)	Cleaned sludge (*n* = 10)	Raw sludge (*n* = 5)	Matrix signal suppression (%)
MeP	209/224	0.47	0.20	57
EtP	193/223	0.18	0.08	56
PrP	193/195	9.26	4.28	53
BuP	195/223	0.19	0.10	47

**Fig. 5:**
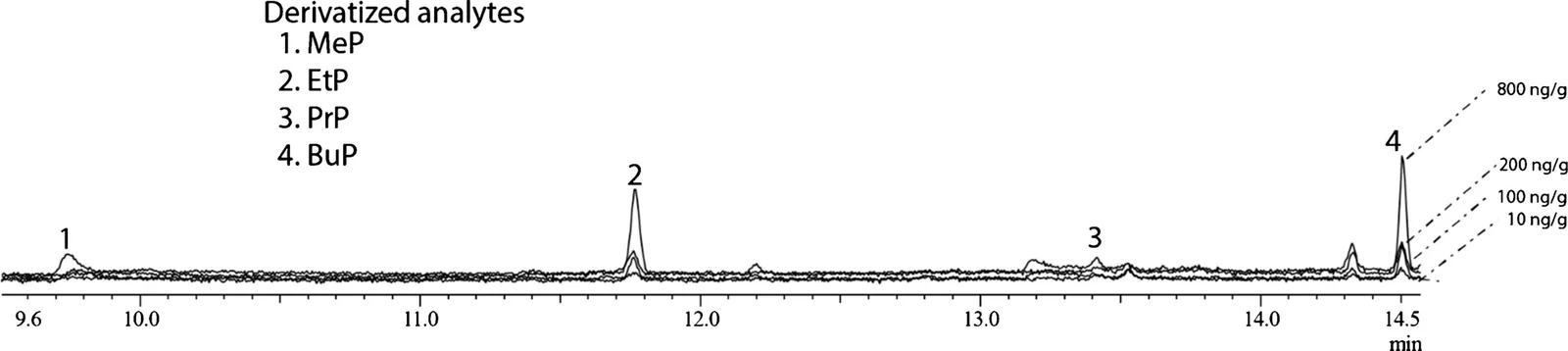
Chromatograms of some calibration curve points over matrix (cleaned sludge from hospital WWTP). Standard addition concentrations: 10, 100, 200, and 800 ng/g

The method exhibited excellent linearity over a dynamic range of 10 to 1000 ng/g, with determination coefficients (*R*^2^) > 0.99 for all analytes, demonstrating that standard additions effectively compensate for matrix suppression (Fig. 5). The choice of monitoring the specific SIM ions—*m/z* 224 and 209 for MeP-TMS; *m/z* 223 and 193 for EtP-TMS; *m/z* 195 and 193 for PrP-TMS; and *m/z* 210 and 195 for BuP-TMS—was technically dictated by their high abundance in the electron ionization (EI) mass spectra, ensuring optimal sensitivity. Under EI conditions, the molecular ions of the heavier parabens (PrP-TMS, *m/z* 252; BuP-TMS, *m/z* 266) suffer from severe fragmentation and exhibit extremely low abundance. Instead, the monitored ions correspond to highly characteristic fragments and rearrangements: *m/z* 193 represents the universal trimethylsilyloxybenzoyl cation *[TMSO-C_6H_4-CO]^*+; *m/z* 210 for BuP-TMS corresponds to the trimethylsilyloxybenzoic acid radical cation *[TMSO-C_6H_4-COOH]^*+ formed via a McLafferty-type rearrangement with the loss of butene (56); and *m/z* 195 represents the fragment *[TMSO-C_6H_4-COOH - CH_3]^*+ formed by the subsequent loss of a methyl radical from the silylated benzoic acid intermediate. This ion selection provides both maximum signal-to-noise ratio and excellent selectivity in the complex sludge matrix.

**Fig. 6:**
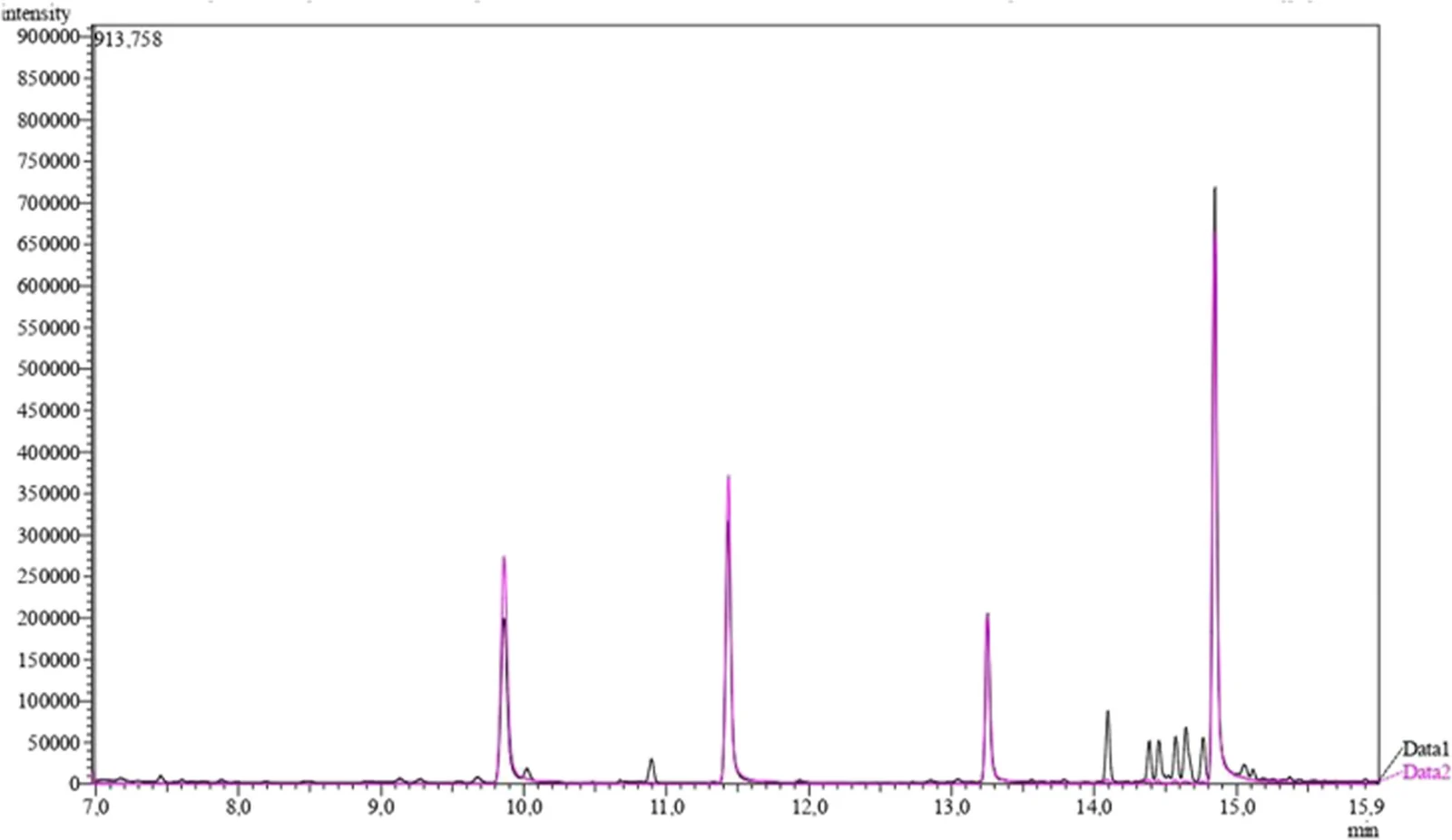
SIM mode chromatogram for parabens of a real (Data 1) and spiked sludge (Data 2) at 800 ng/g extraction using DVB/CAR/PDMS SPME fiber after RDSE leaching

The limits of detection (LOD) and limits of quantification (LOQ) were calculated based on the signal-to-noise ratio (S/N) of 3 and 10, respectively, from blank baseline noise. The method showed low detection limits (LODs) ranging from 2.1 ng/g (PrP) to 5.2 ng/g (MeP). Method repeatability and reproducibility were evaluated using three independent replicates (*n* = *3*), yielding relative standard deviations (RSD) below 9%. Furthermore, accuracy was assessed through recovery tests on spiked clean sludge matrices at a concentration of 800 ng/g (Fig. 6). Although a high concentration of 800 ng/g was selected to ensure signal robustness for precision and accuracy measurements, the method’s accuracy was also successfully verified at lower levels (50 ng/g) during daily standard additions calibration, yielding comparable recoveries (82–95%). This confirms that the partition-based extraction remains highly efficient across the entire analytical range without saturation of the active sites. The recovery rates ranged from 80 to 96%, demonstrating that the RDSE-SPME approach is both highly reproducible and accurate for these targets (Table 3).

**Table 3:** SIM conditions and analytical validation characteristics of the developed method. *TMS derivative of the analyte (mono-TMS)

Compound*	tR (min)	Quant Ion (m/z)	Qual Ion (m/z)	Slope	Intercept	R^2^	Recovery (%)	RSD (%)	LOD (ng/g)	LOQ (ng/g)	Quantified Amount (ng/g)
MeP	9.875	209	224	1.5637	343	0.9989	95	6.2	4.0	13.3	< LOQ
EtP	11.447	193	223	0.5629	501	0.9938	96	7.9	3.6	12.0	< LOQ
PrP	13.264	193	195	0.1853	91	0.9973	88	7.6	5.2	17.3	20.0 ± 2.4
BuP	14.856	195	210	1.0166	15	0.9989	80	8.3	2.1	7.0	< LOQ

### Analysis of real sludge samples

**Fig. 7:**
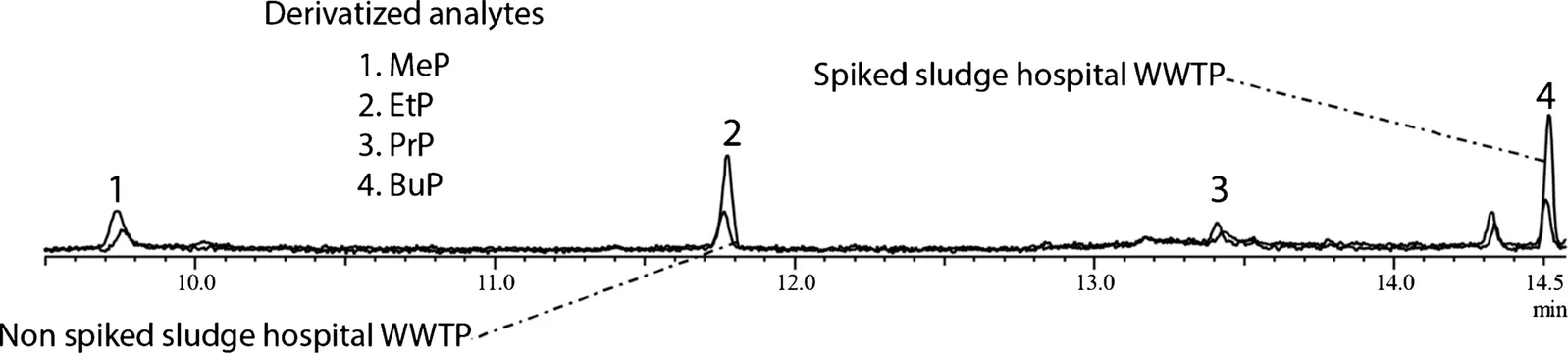
Recovery proof using real (raw) sludge from hospital WWTP. Comparison of unspiked real sample (Data 1) and standard addition spiked matrix (Data 2)

The validated methodology was applied to determine paraben concentrations in raw sludge obtained from the hospital WWTP. The significant matrix effect observed in the raw sewage sludge (approximately 50% signal suppression, Table 2) is attributed to co-extracted dissolved organic carbon (DOC) and humic substances that permeate the filter membrane, competing for SPME fiber adsorption sites or causing signal attenuation. In addition, the single hospital sludge sample analyzed represents a proof of concept, and future systematic monitoring will be required to characterize temporal and spatial variability. To accurately quantify the parabens and overcome this severe signal suppression without resorting to intensive, solvent-consuming clean-up steps, a standard additions protocol was rigorously applied. Known standard concentrations were sequentially added to aliquots of the real sludge sample, and the endogenous concentrations were determined by extrapolating the linear regression to the x-axis. Using this approach, all four parabens were confirmed in the samples. MeP, EtP, and BuP were detected below their limits of quantification (< LOQ), while PrP was quantified at 20.0 ± 2.4 ng/g (where the uncertainty represents the expanded uncertainty U with a coverage factor k = 2, corresponding to a 95% confidence level, derived from the standard additions regression analysis). While the standard additions method increases the number of chromatographic runs per sample, the overnight leaching step can be performed in parallel for up to 12 or more samples using multi-position magnetic stirrers. Thus, the active operator/instrument time remains under 60 min per sample, making the workflow highly feasible for routine monitoring once the parallel equilibration is complete. This confirms that a bioavailable fraction of these endocrine disruptors is present and can potentially be released into the environment (Fig. 7).

### Advantages, disadvantages, and method comparison

The proposed RDSE-SPME methodology presents significant **advantages** for environmental monitoring. First, it uniquely targets the *bioavailable fraction* of pollutants by simulating aqueous leaching, which provides more relevant toxicological data than total exhaustive extraction. Second, the combination of RDSE and SPME eliminates the need for multiple manual clean-up steps and highly toxic organic solvents (like methanol or dichloromethane) typically required in conventional sludge analyses. However, a notable **disadvantage** is the unavoidable need for a derivatization step prior to GC-MS analysis, which adds time and requires handling of MSTFA, as well as the presence of matrix effects that necessitate the use of the standard additions method. Nevertheless, the manual handling of the SPME fiber transfer and on-fiber silylation can be easily automated in modern laboratories using commercial robotic autosamplers (e.g., dual-head autosamplers), which would completely eliminate manual handling and further enhance reproducibility.

Compared to reported approaches such as SPE [21, 22], MSPD, or conventional SBSE [18] applied to complex matrices, our method offers highly competitive detection limits (2.1–5.2 ng/g compared to typical 5–20 ng/g ranges reported in literature). Moreover, while traditional extraction from sludge requires grams of sample and tens of milliliters of organic solvents, the RDSE approach utilizes merely 40 mg of solid sample and extracts directly into water (Table 4).

**Table 4:** Comparison of the proposed RDSE-SPME method with other analytical methodologies for the determination of parabens in environmental solid matrices

Method	Sample Mass (g)	Organic Solvent (mL)	Extraction Time (h)	LOD (ng/g)	Recovery (%)	AGREEprep	BAGI	VIGI
SPE [21]	1.0	15.0	2.5	5.0–15.0	78–92	0.28	45.0	35
MSPD [22]	0.5	8.0	1.5	4.0–10.0	81–94	0.32	50.0	40
SBSE [18]	0.2	5.0	1.0	3.0–8.0	84–95	0.45	55.0	48
Proposed RDSE-SPME	0.04	0.02 (MSTFA)	0.5 (active)	2.1–5.2	80–96	0.54	75.0	75

### Method greenness, practicality, and innovation assessment

**Fig. 8:**
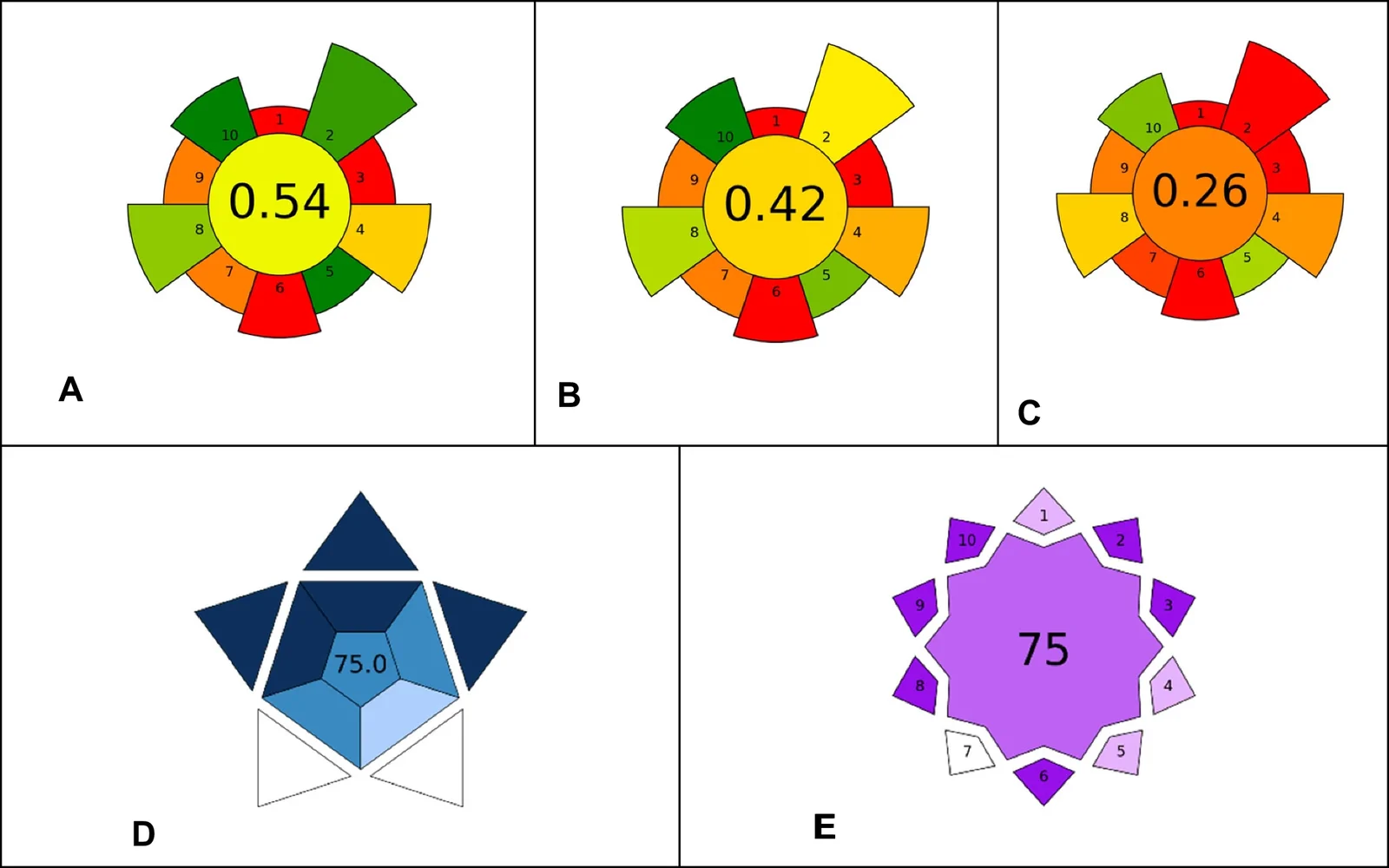
Comparison of the greenness assessment results on the **A** SPME, **B** SBSE, and **C** MSPD methods used for screening parabens in sludge from WWTP after applying the default weights. Also shown are the **D** BAGI applicability, and **E** VIGI innovation metrics of the newly developed analytical method RDSE-SPME

To establish the credentials of the developed RDSE-SPME method, a comprehensive evaluation using green, blue, and violet metrics was performed (Fig. 8). The environmental footprint of the sample preparation step was formally assessed using the AGREEprep tool, yielding a commendable score of 0.54 (Fig. 8a–c). This score reflects the minimized sample mass (40 mg, satisfying the < 0.5 g criterion), the use of water as the primary extraction solvent avoiding toxic organic waste, and the energy-efficient, room-temperature on-fiber derivatization step. The main penalty arises from the chemical derivatization step (MSTFA) intrinsic to GC-MS analysis of polar compounds. It should be noted that the exhaustive cleaning protocol described in is not factored into the AGREEprep score, as it is a one-time procedure for reference material preparation rather than a step in the routine analytical workflow. Input details and parameters for greenness, practicality, and innovation score models are fully detailed in Tables S1–S3 of the Electronic Supplementary Material (ESM).

Practical applicability and routine feasibility were evaluated using the BAGI tool, resulting in a high score of 75.0 (out of 100), which significantly exceeds the practicality threshold of 60.0. The corresponding blue star pictogram (Fig. 8d) highlights the method’s strong operational advantages: five attributes scored maximum practicality (dark blue, 10 points each), including the quantitative/confirmatory analysis type, simultaneous preparation of multiple samples (up to 12 or more using a multi-position magnetic stirrer), low sample amount requirements (40 mg), and the use of simple, commercially available reagents. Three attributes scored intermediate practicality (light blue, 5 points each), including the multi-residue extraction of 4 analytes of the same class, the use of standard benchtop GC-MS instrumentation, and the one-step preconcentration nature of SPME. The main limitations (white, 2.5 points each) relate to the manual handling of the extraction and fiber desorption, which is not fully automated.

Lastly, the innovative character of the method was evaluated using the VIGI tool, obtaining a score of 75 (out of 100), well above the innovation threshold of 50. The violet decagon pictogram (Fig. 8e) shows that six out of ten criteria achieved high innovation (dark violet, 10 points each): data processing/software (incorporating chromatographic simulation and advanced NIST data processing), White Analytical Chemistry principles (integrating AGREEprep, BAGI, and VIGI), miniaturization (micro-scale rotating disk and fiber extraction), interdisciplinarity (applicable to environmental monitoring, hospital waste, and toxicology), sensitivity (low ng/g detection limits), and conceptual approach (a novel strategy targeting the leached bioavailable fraction of parabens in sludge instead of total exhaustive concentration). Three criteria achieved moderate innovation (light violet, 5 points each): advanced sample preparation (strongly agreeing on SPME use but with manual steps), regulatory compliance (addressing EPA/EU guidelines on emerging contaminants), and innovative materials (relying on commercial polymer coatings). The only non-innovative parameter (white, 0 points) was the automation grade, as the current extraction is performed manually. Collectively, the high AGREEprep, BAGI, and VIGI scores demonstrate that the developed RDSE-SPME methodology represents a highly sustainable, practically viable, and cutting-edge analytical solution for environmental monitoring.

## Conclusions

As a proof-of-concept study (building upon our pioneering work using this method on natural sludge, see Ref. [16]), this methodology has certain structural limitations that should be noted. The exhaustive matrix cleaning procedure used for validation may not entirely reflect the physical-chemical entrapment of parabens in unaltered natural sludge, potentially influencing recovery rates in real scenarios. Furthermore, the application to real samples was limited to a single specimen where three out of four analytes were detected below the limit of quantification (LOQ). Finally, the severe matrix effects observed—which reduced the signal by approximately 50%—confirm the strong interaction of the analytes with the matrix components. This ratifies the absolute dimension and necessity of using strategies for correcting matrix effects, such as the standard additions method applied here, although the future incorporation of isotopically labeled internal standards is highly recommended for routine applications to further enhance accuracy and precision.

The hyphenated reverse-RDSE-SPME-GC-MS method represents a sustainable and innovative solution for analyzing the bioavailable fraction of parabens in sewage sludge. Under optimized conditions, the method achieved low detection limits (2.1–5.2 ng/g) and recoveries (80–96%) using only 40 mg of sample and avoiding organic solvents during extraction. The green, practical, and innovative credentials of the method were confirmed by AGREEprep (0.54), BAGI (75.0), and VIGI (75) scores, demonstrating compliance with White Analytical Chemistry. In the real hospital sludge sample, PrP was quantified at 20.0 ± 2.4 ng/g, whereas MeP, EtP, and BuP were detected below their limits of quantification (< LOQ). These detected levels lie well below typical predicted no-effect concentrations (PNEC) in soil (e.g., > 1000 ng/g), indicating that the immediate ecotoxicological risk of this bioavailable fraction is low. However, the presence of these compounds highlights the need for continued monitoring using green and practical tools.

## Supplementary Information

Below is the link to the electronic supplementary material.

ESM 1 (DOCX 31.6 KB)

## Data Availability

The datasets generated and analyzed during the current study are available from the corresponding author upon reasonable request.

## Abbreviations

*AGREEprep*: Analytical Greenness Metric for Sample Preparation
*BuP*: Butylparaben
*CAR*: Carboxen
*DI*: Direct immersion
*DVB*: Divinylbenzene
*EtP*: Ethylparaben
*GC–MS*: Gas chromatography-mass spectrometry
*HS*: Headspace
*LOD*: Limit of detection
*LOQ*: Limit of quantification
*MeP*: Methylparaben
*MSPD*: Matrix solid-phase dispersion
*MSTFA*: N-Methyl-N-(trimethylsilyl)trifluoroacetamide
*m/z*: Mass to charge ratio
*NIST*: National Institute of Standards and Technology
*PA*: Polyacrylate
*PDMS*: Polydimethylsiloxane
*PrP*: Propylparaben
*RDSE*: Rotating disk sorptive extraction
*RSD*: Relative standard deviation
*SBSE*: Stir bar sorptive extraction
*SIM*: Selected ion monitoring
*S/N*: Signal to noise ratio
*SPE*: Solid-phase extraction
*SPME*: Solid-phase microextraction
*TOC*: Total organic carbon
*WWTP*: Wastewater treatment plant

## References

[1] Boberg J, Taxvig C, Christiansen S, Hass U. Possible endocrine disrupting effects of parabens and their metabolites. Reprod Toxicol. 2010;30:301–12. 10.1016/j.reprotox.2010.03.011. 20381602

[2] Lundov MD, Moesby L, Zachariae C, Duus JB. Contamination versus preservation of cosmetics: a review on legislation, usage, infections, and contact allergy. Contact Derm. 2009;60:70–8. 10.1111/j.1600-0536.2008.01501.x. 19207376

[3] Meyer BK, Ni A, Hu B, Shi L. Antimicrobial preservative use in parenteral products: past and present. J Pharm Sci. 2007;96:3155–67. 10.1002/jps.20976. 17722087

[4] Błędzka D, Gromadzińska J, Wąsowicz W. Parabens: from environmental studies to human health. Environ Int. 2014;67:27–42. 10.1016/j.envint.2014.02.007. 24657492

[5] Darbre PD, Harvey PW. Paraben esters: review of recent studies of endocrine toxicity, absorption, esterase and human exposure. J Appl Toxicol. 2008;28:561–78. 10.1002/jat.1358. 18484575

[6] Oishi S. Effects of propyl paraben on the male reproductive system. Food Chem Toxicol. 2002;40:1807–13. 10.1016/S0278-6915(02)00204-1. 12419695

[7] Taxvig C, Vinggaard AM, Hass U, Nellemann C, Metzdorff GE, Hansen PR. Do parabens have the ability to interfere with steroidogenesis? Toxicol Sci. 2008;106:206–13. 10.1093/toxsci/kfn148. 18648085

[8] Eriksson E, Andersen HR, Ledin A. Substance flow analysis of parabens in Denmark complemented with a survey of presence and frequency in various commodities. J Hazard Mater. 2008;156:240–59. 10.1016/j.jhazmat.2007.12.022. 18243542

[9] Anderson FA. Final amended report on the safety assessment of methylparaben, ethylparaben, propylparaben, isopropylparaben, butylparaben, isobutylparaben, and benzylparaben as used in cosmetic products. Int J Toxicol. 2008;27:1–82. 10.1080/10915810802548359. 19101832

[10] Honda M, Robinson M, Kannan K. Parabens in human urine from several Asian countries, Greece, and the United States. Chemosphere. 2018;201:13–9. 10.1016/j.chemosphere.2018.02.165. 29510318

[11] Soni MG, Carabin IG, Burdock GA. Safety assessment of esters of p-hydroxybenzoic acid (parabens). Food Chem Toxicol. 2005;43:985–1015. 10.1016/j.fct.2005.01.020. 15833376

[12] Camino-Sánchez FJ, Zafra-Gómez A, Dorival-García N, Juárez-Jiménez B, Ruano-Caballero AM, Vílchez JL. Determination of selected parabens, benzophenones, triclosan and triclocarban in agricultural soils. Talanta. 2016;150:415–24. 10.1016/j.talanta.2015.12.031. 26838425

[13] Richter P, Leiva C, Choque C, Giordano A, Restrepo-Parra E. Rotating-disk sorptive extraction of nonylphenol from water samples. J Chromatogr A. 2009;1216:8598–602. 10.1016/j.chroma.2009.10.044. 19879588

[14] Velasco E, Ríos-Acevedo JJ, Sarria-Villa R, Rosero-Moreano MH. Green method to determine triazine pesticides in water using rotating disk sorptive extraction (RDSE). Heliyon. 2021;7:e07878. 10.1016/j.heliyon.2021.e07878. PMC8426532 34522798

[15] Cañas A, Valdebenito S, Richter P. A new rotating-disk sorptive extraction mode. Anal Bioanal Chem. 2014;406:2205–10. 10.1007/s00216-014-7628-8. 24518899

[16] Mendes LD, Frena M, Madureira LAS, Rosero-Moreano M, Richter P, Carasek E. Eco-friendly method for PCBs in solid waste by GC–MS. J Environ Chem Eng. 2018;6:5042–8. 10.1016/j.jece.2018.07.037.

[17] Becerra-Herrera M, Miranda V, Arismendi D, Richter P. Chemometric optimization of the extraction and derivatization of parabens for their determination in water samples by rotating-disk sorptive extraction and GC–MS. Talanta. 2018;176:551–7. 10.1016/j.talanta.2017.08.071. 28917789

[18] Ferreira AM, Möder M, Laespada ME. Stir bar sorptive extraction of parabens. J Chromatogr A. 2011;1218:3837–44. 10.1016/j.chroma.2011.04.055. 21571279

[19] Bian L, Li S, Ge X, Zhou Y, Zhang J. Determination of bisphenols, triclosan, and parabens in bottled water by solid-phase microextraction combined with GC–MS/MS and assessment of the associated health risk. J Food Compos Anal. 2023;123:105548. 10.1016/j.jfca.2023.105548.

[20] Argenta TS, Barros ARM, de Carvalho CAAS, de Oliveira T, Tonetti AL. Parabens in aerobic granular sludge systems. Sci Total Environ. 2021;753:142105. 10.1016/j.scitotenv.2020.142105. 33207471

[21] Chen J, Pycke BFG, Brownawell BJ, Halden RU. Occurrence and temporal variation of five parabens in sewage sludge. Sci Total Environ. 2017;593–594:368–74. 10.1016/j.scitotenv.2017.03.162. PMC5510738 28346910

[22] Albero B, Pérez RA, Sánchez-Brunete C, Tadeo JL. Parabens in sewage sludge from Madrid. J Hazard Mater. 2012;239–240:48–55. 10.1016/j.jhazmat.2012.05.017. 22640822

[23] Raza N, Kim KH, Abdullah M, et al. Quantitation approaches for parabens in human samples. TrAC Trends Anal Chem. 2018;98:161–73. 10.1016/j.trac.2017.11.009.

[24] Saraji M, Ghani M. HF-LLLME followed by SPME for chlorophenols. J Chromatogr A. 2015;1418:45–53. 10.1016/j.chroma.2015.09.062. 26411480

[25] Serna-Galvis EA, Botero-Coy AM, Rosero-Moreano M, Torres-Palma RA. An initial approach to the presence of pharmaceuticals in wastewater from hospitals in Colombia and their environmental risk. Water. 2022;14:950. 10.3390/w14060950.

[26] Wojnowski W, Tobiszewski M, Pena-Pereira F, Psillakis E. AGREEprep – analytical greenness metric for sample preparation. TrAC Trends Anal Chem. 2022;149:116553. 10.1016/j.trac.2022.116553.

[27] Wojnowski W, Tobiszewski M, Pena-Pereira F, Psillakis E. Blue applicability grade index (BAGI) and software: a new tool for the evaluation of method practicality. Green Chem. 2023;25:100–12. 10.1039/D2GC03831B.

[28] Fuente-Ballesteros A, Martínez-Martínez V, Ares AM, Valverde S, Samanidou V, Bernal J. Violet innovation grade index (VIGI): A new survey-based metric for evaluating innovation in analytical methods. Anal Chem. 2025;97:6946–55. 10.1021/acs.analchem.4c04221. PMC12131222 40139928

