# Supplementary material for: Eco-friendly method for the analysis of the soluble fraction of parabens leached from sludge of a hospital wastewater treatment plant—proof of concept
Source: Anal Bioanal Chem. 2026 Oct 8;418(22):7327–38. doi: 10.1007/s00216-026-06762-1 (PMC13736439; doi:10.1007/s00216-026-06762-1)
Supplement: Supplementary file 1 [file 216_2026_6762_MOESM1_ESM.docx]

ELECTRONIC SUPPLEMENTARY MATERIAL (ESM)

Eco-friendly method for the analysis of the soluble fraction of parabens leached from sludge of a hospital wastewater treatment plant - proof of concept

S. Bedoya Patiño¹, J.P. Arrubla Vélez³, J.M. Grisales Bedoya³, M. Becerra-Herrera⁴, P. Richter⁵, E. Carasek⁶, J.J. Ríos Acevedo⁷, M. Rosero-Moreano²*

*Corresponding Author: M. Rosero-Moreano	Email: milton.rosero@ucaldas.edu.co

____________________________________________________

SECTION S1. SAMPLE CONDITIONING AND MATRIX CLEAN-UP FLOW

**Figure S1. Hospital Santa Sofia’s wastewater treatment plant, collecting and conditioning sludge samples steps:** A) Activated sludge mechanism from sediment tank sample site; B) Fresh sludge WWTP in laboratory for pH and temperature control; C-D) Cleaning steps using solvent liquid extraction; E-F) Ground and sieved sludge ready for analysis.

SECTION S2. GREENNESS COMPARISON PARAMETERS (AGREEprep)

**Table S1. Input parameters and ratings for AGREEprep greenness metric comparison of the proposed RDSE-SPME method, SBSE method [18], and MSPD method [22].**

| **AGREEprep Principle** | **Proposed RDSE-SPME** | **SBSE Method [18]** | **MSPD Method [22]** |
| --- | --- | --- | --- |
| 1. Placement of sample prep | Ex-situ (Analytical laboratory) (Score: 0.5) | Ex-situ (Analytical laboratory) (Score: 0.5) | Ex-situ (Analytical laboratory) (Score: 0.5) |
| 2. Device & sample mass | Miniaturized disk, 40 mg sample (Score: 1.0) | Stir bar extraction, 200 mg sample (Score: 0.8) | Mortar grinding, 500 mg sample (Score: 0.4) |
| 3. Reagents & solvents | Water leaching, 20 µL MSTFA (Score: 0.9) | Water extraction, organic cleaning (Score: 0.6) | High organic solvent volume (Score: 0.3) |
| 4. Waste generation | Very low (<0.1 mL liquid waste) (Score: 1.0) | Low (~5 mL liquid waste) (Score: 0.7) | Medium (>15 mL organic waste) (Score: 0.4) |
| 5. Energy consumption | Low (room-temp extraction/deriv.) (Score: 0.8) | Medium (thermal desorpt. heating) (Score: 0.6) | Medium (evaporation heating) (Score: 0.5) |
| 6. Sample throughput | High (up to 12+ samples in parallel) (Score: 0.8) | Medium (limited batch handling) (Score: 0.6) | Low (sequential manual elution) (Score: 0.4) |
| 7. Integration of operations | High (combined leaching-extraction) (Score: 0.8) | Medium (separate wash step required) (Score: 0.6) | Low (elution-evap-deriv. series) (Score: 0.3) |
| 8. Operator safety | High (closed vial silylation) (Score: 0.9) | Medium (solvent wash handling) (Score: 0.7) | Low (high exposure to DCM/MeOH) (Score: 0.3) |
| 9. Renewable materials | Medium (reusable disks, paper filters) (Score: 0.5) | Medium (reusable stir bars) (Score: 0.5) | Low (disposable plastic cartridges) (Score: 0.2) |
| 10. Solvent recyclability | N/A (water-based extraction) (Score: 0.5) | N/A (water-based extraction) (Score: 0.5) | Low (solvents disposed of directly) (Score: 0.1) |
| Overall AGREEprep Score | 0.54 (Greener) | 0.45 (Moderate) | 0.32 (Poor) |

SECTION S3. PRACTICALITY BREAKDOWN (BAGI)

**Table S2. Detailed scoring breakdown for BAGI (Blue Applicability Grade Index) for the proposed RDSE-SPME method (Overall Score = 75.0).**

| **BAGI Attribute** | **Score** | **Method Feature & Justification** |
| --- | --- | --- |
| 1. Type of analysis | 10.0 (Dark Blue) | Quantitative and confirmatory analysis. Separation, quantification, and MS spectra fragment matching. |
| 2. Number of analytes | 5.0 (Light Blue) | Multi-residue determination targeting 4 analytes (MeP, EtP, PrP, BuP) of the same chemical class. |
| 3. Instrumentation | 5.0 (Light Blue) | Sophisticated desktop instrumentation (GC-MS) commonly available in modern testing laboratories. |
| 4. Parallel treatment | 5.0 (Light Blue) | Simultaneous preparation of up to 12 samples using a multi-position magnetic stirrer during overnight leaching. |
| 5. Sample preparation scale | 5.0 (Light Blue) | Miniaturized extraction technique (solid-phase microextraction, SPME) coupled to rotating disk leaching. |
| 6. Sample throughput | 5.0 (Light Blue) | Throughput of 2–4 samples per hour (active chromatography run time of ~15 min). |
| 7. Reagents and materials | 7.5 (Blue) | Commercially available reagents (MSTFA) and materials (reusable Teflon disks, commercial fibers) requiring no synthesis. |
| 8. Preconcentration | 7.5 (Blue) | Preconcentration achieved in a single step (direct extraction of the leached analytes onto the SPME fiber). |
| 9. Automation degree | 2.5 (White) | Manual transfer of the SPME fiber from extraction to derivatization and injection (not automated). |
| 10. Amount of sample | 10.0 (Dark Blue) | Very low sample amount (40 mg of solid sludge sample), satisfying the threshold of <10 g for environmental matrices. |
| Overall BAGI Score | 75.0 (Practical) | Calculated score exceeds the practicality threshold (score >= 60.0) for routine quality control labs. |

SECTION S4. INNOVATION BREAKDOWN (VIGI)

**Table S3. Detailed scoring breakdown for VIGI (Violet Innovation Grade Index) for the proposed RDSE-SPME method (Overall Score = 75.0).**

| **VIGI Criterion** | **Score** | **Method Feature & Justification** |
| --- | --- | --- |
| 1. Sample prep. & instrumentation | 10.0 (Dark Violet) | Combines advanced sample preparation methods (RDSE in reverse mode and SPME) and GC-MS. |
| 2. Data processing & software | 10.0 (Dark Violet) | Incorporate chromatographic simulation (GC-MS simulation) and advanced NIST 14 database match (>85%). |
| 3. White analytical chemistry | 10.0 (Dark Violet) | Integrates multiple greenness, practicality, and innovation metrics (AGREEprep, BAGI, VIGI) to evaluate WAC. |
| 4. Regulatory compliance | 5.0 (Light Violet) | Addresses emerging pollutant monitoring (parabens) in environmental waste, complying with EPA/EU recommendations. |
| 5. Materials and reagents | 5.0 (Light Violet) | Relies on commercial polymeric fiber coatings (DVB/CAR/PDMS) and specialized Teflon rotating disk assemblies. |
| 6. Miniaturization | 10.0 (Dark Violet) | Highly miniaturized extraction device (40 mg sludge sample and micro-scale SPME fiber). |
| 7. Automatization grade | 0.0 (White) | Entire workflow (extraction, derivatization, and injection) is performed manually in the current setup. |
| 8. Interdisciplinarity | 10.0 (Dark Violet) | Applicable across environmental monitoring, ecotoxicology, wastewater engineering, and clinical toxicology. |
| 9. Sensitivity | 5.0 (Light Violet) | Achieves low ng/g limits of detection (2.1–5.2 ng/g), which are highly competitive for complex sewage sludge. |
| 10. Approach | 10.0 (Dark Violet) | Highly innovative conceptual approach targeting the soluble, bioavailable fraction leached instead of total extraction. |
| Overall VIGI Score | 75 (Innovative) | Calculated score exceeds the innovation threshold (score >= 50) for newly developed analytical methods. |
